# Menstruation and sexual health, well-being and justice

**DOI:** 10.2471/BLT.23.291159

**Published:** 2024-10-29

**Authors:** Carmen H Logie, Inga T Winkler, Manjulaa Narasimhan, Amita Bhakta, Tara Hutchingame, Tahirah Reynolds, Ursula Maschette, Scheaffer Okore, Sarah Njoki W Ngumi, Dani J Barrington

**Affiliations:** a Factor-Inwentash Faculty of Social Work, University of Toronto, 246 Bloor Street W, Room 504, Toronto, M5S 1V4 Ontario, Canada.; b Department of Social Sciences, Wageningen University, Wageningen, Kingdom of the Netherlands.; c Department of Sexual and Reproductive Health and Research, World Health Organization, Geneva, Switzerland.; d Leicester, England.; e São Paulo, Brazil.; f The Venio Group, Nairobi, Kenya.; g School of Population and Global Health, The University of Western Australia, Crawley, Australia.

While early efforts by researchers, advocates and practitioners on improving menstrual health focused on menstrual hygiene management, recent initiatives have advanced an understanding of menstrual health^1^ within a comprehensive approach to sexual and reproductive health and rights.^2^ This understanding includes recognition of both the biological linkages between menstrual health and sexual and reproductive health and rights, such as the impact of menstrual disorders on reproductive health, and sociocultural linkages such as the impact of menstruation-related stigma on socioeconomic outcomes and shame. Yet, to date researchers’ emphasis has been on establishing the linkages with reproductive health^3^ to the neglect of sexual health – the focus of our article.

The World Health Organization’s framework for operationalizing sexual health^3^ acknowledges linkages between the sexual health and reproductive health domains, such as contraception provision. Extending the sexual health framing to menstruation facilitates a holistic examination of the menstrual cycle. Sexual health, sexual well-being and sexual justice all interact and offer insights into better understanding and advancing menstrual health across the life course. Sexual well-being domains relevant to menstruation include sexual respect, sexual self-esteem and comfort with sexuality.^4^ Sexual justice represents larger global efforts to ensure social, cultural and legal support for equitable, person-centred sexual and reproductive health.^4^ Sexual rights, as a component of sexual justice, include the realization of human rights with respect to one’s sexuality and sexual health, including protection from stigma and discrimination.^3^ Furthermore, a life course perspective includes recognition of biological, behavioural and psychological processes that operate across an individual’s life,^5^ and this perspective has not been adequately integrated in menstrual research, policy, education and advocacy. In this article, we contextualize menstruation in relation to sexual health, sexual well-being and sexual justice across the life course, and argue that these dimensions are essential to overall health and well-being outcomes.

## Sexual health and menstruation

Sexual health integrates multiple domains pertinent to menstrual health, including comprehensive sexuality education, sexual function and psychosocial counselling.^3^ As menstruation-related misconceptions (such as the unfounded belief that menstruation is dirty) are common across global contexts, comprehensive sexuality education can play a fundamental role in providing skills (for example, self-management), knowledge and information on the menstrual cycle, potentially reducing menstrual stigma.^6^ However, where sexuality education takes place, it often does not cover the menstrual cycle, and where specific menstrual education programmes exist, they frequently only cover menstrual hygiene. Moreover, programmes focus primarily on menarche, with little, if any, discussion of menstrual health in adult life and the menopause transition. In high-income countries, many studies have identified a lack of menstrual literacy not only in those who menstruate, but also in their networks.^7^ This knowledge gap points to the need for comprehensive sexuality education that advances menstrual literacy for people across age brackets and genders. Menstrual health can be integrated into comprehensive sexuality education for youth, caregivers, parents and communities, and should include supporting health and care workers to address physiological and sociocultural dimensions of the menstrual cycle.^2^ Comprehensive sexuality education that emphasizes agency, self-esteem and healthy relationships in relation to menstrual health can reduce stigma and contribute to well-being during the entire menstrual cycle and across the life course.

Sexual health also includes psychosocial counselling to address interacting physiological, psychological and relational factors for sexual concerns, difficulties, dysfunctions and disorders.^3^ Addressing menstrual health could include psychosocial counselling for menstrual disorders and concerns that constrain daily life activities^2^ such as dysmenorrhea (pain during menstruation), menorrhagia (heavy menstrual bleeding) and endometriosis (growth of tissue similar to the uterus lining outside of the uterus). While fertility is a major concern with some menstrual health conditions, many individuals also prioritize their wider quality of life, which also needs to inform medical care. In addition, psychological counselling may be helpful during perimenopause to offer support and empowerment for self-care activities to realize optimal health during this life course transition, in addition to providing community-level awareness and engagement to meet social and health needs.^8^ Integrating the full life course of menstruation into psychosocial elements of sexual health provides an opportunity to improve well-being beyond the narrow focus on reproduction.

## Sexual well-being and menstruation

Menstrual stigma can threaten several facets of sexual well-being. For instance, menstrual stigma reduces the ability to realize sexual respect (referring to the perceived positive regard by others for one’s sexual personhood);^4^ sexual self-esteem (the positive regard of oneself and one’s body sexually); and comfort with sexuality (referring to comfort, ease and a lack of shame in communicating about and attending to sexual health practices).^4^ Stigma towards menstruation is rooted in deep-seated societal, cultural and religious values and beliefs prevalent across the globe that perceive menstruation as polluting and dangerous. Systematic reviews on menstrual experiences^7^^,^^9^ reveal complex processes of menstrual stigma. These reviews conceptualize pathways through which sociocultural contexts of menstrual stigma contribute to internally and externally enforced behavioural expectations, which in turn shape confidence and contribute to negative emotional responses such as shame, embarrassment, disgust or psychosocial distress. Menstrual stigma^7^^,^^9^ can stigmatize sexual activity during menstruation and result in avoiding seeking sexual pleasure. This avoidance may be attributed to internalized stigma by the person menstruating as well as perceived stigma by sex partners towards menstrual blood.

The life course perspective is also relevant to understanding stigma, as integrating timescales into stigma research can provide insight into how experiences change across human developmental phases.^10^ Menstrual stigma shifts in its manifestations and impacts across menstruation stages from menarche in adolescence to adulthood and into perimenopause and post-menopause. Timescale approaches also examine how stigma changes across historical contexts.^10^ Doing so is relevant to understanding menstrual stigma, as people reaching menarche since the late 20th century may be more likely to challenge the secrecy of menstruation and engage boys and men in menstruation discussions.^7^^,^^11^ This shift reflects broader societal changes towards acknowledging menstruation as a natural part of life that signals reduced menstruation-related stigma over time.^11^

While menstrual stigma remains pervasive across global contexts, individuals and groups are increasingly challenging stigma and enact agency in the context of menstruation, including, for instance, through normalizing discussions about menstrual sex. However, given that stigma is structural, the expectation of reducing it cannot be on menstruators alone. Frameworks such as sexual well-being can be applied to inform targeted menstrual stigma reduction interventions that address stigma drivers (such as misinformation) across the life course among individuals, families and communities.

## Sexual justice and menstruation

Sexual justice includes trauma-informed practices that consider adverse sexual experiences across the life course, person-centred approaches, and a focus on equity and human rights that are key to realizing sexual and reproductive health.^4^ The concepts of sexual justice and menstrual justice are complementary. Sexual justice is key to the realization of optimal sexual health and sexual well-being, and similarly, menstrual justice is a core component of realizing menstrual health. Menstrual injustice can be understood as the oppression of menstruators, women, girls, transgender men and boys, and nonbinary people, simply because they menstruate.^12^ Menstrual justice thus considers menstrual experiences beyond those linked to reproduction. Menstrual justice includes access to menstrual products, water and sanitation resources and infrastructure, and menstrual health services. However, this justice extends beyond material needs to call attention to larger social contexts of inequity, stigma, coercion, violence and discrimination that shape menstrual experiences. Menstrual justice recognizes menstrual health as a human right, but also acknowledges meeting menstrual needs as a prerequisite for realizing other human rights, including access to education, public space, dignity and autonomy.

Justice perspectives draw particular attention to intersectional forms of stigma and discrimination. People experience menstrual stigma, discrimination and injustices differently due to additional and intersecting stressors such as poverty, and reduced access to social, health, political and economic resources because of racism, gender discrimination, lesbian, gay, bisexual, transgender and queer stigma, poverty, ableism, incarceration and ageism.^7^^,^^9^ For instance, as experiences of menarche are socially and contextually produced, some non-binary and transgender people who menstruate may feel gender dysphoria (distress due to incongruence between sex assigned at birth and gender identity) after menarche. People with cognitive disabilities may be coerced into menstrual suppression via long-term contraception and sterilization (such as hysterectomy or tubal ligation) to facilitate menstrual management, which affects their sexual health.^13^ Integrating menstrual justice into the larger sexual justice framework holds the potential to identify and subsequently address the diversity of menstrual health needs, equity-related issues and priorities across the life course.

## Conclusions

Menstrual experiences and needs are dynamic across the life course and are linked with sexual health, sexual well-being and sexual justice. Understanding and addressing the complexity and variability in menstrual experiences requires research, policy and practice to apply a life course approach, as well as to consider experiences of intersecting stigma and discrimination targeting socially devalued identities (such as sexism) and experiences (such as menstruation). Such a life course approach to menstruation can provide insight into the ways in which menarche- and menopause-associated norms and rituals are culturally and contextually situated. These contexts, differences and preferences produce a range of experiences of menstruation, all of which are valid.

The integration of menstrual health into sexual health, sexual well-being and sexual justice research, programmes, advocacy and policy offers the possibility to reach shared goals between sexual and reproductive health and rights and menstrual health. This alignment has the potential to contribute to gender justice, improve health and well-being, and advance the realization of human rights.
